# Feasibility and safety of automated chest compression during helicopter rescue with hoisting

**DOI:** 10.1016/j.resplu.2025.101212

**Published:** 2026-01-02

**Authors:** Alexandre Carron, Vivien Brenckmann, Alexandre Behouche, Pierre Bouzat, Lilian Barlet, Juliette Meyzenc, Marc Blancher, Katell Berthelot, Guillaume Debaty, Nicolas Segond

**Affiliations:** a Emergency Department and Mobile Intensive Care Unit, University Hospital of Grenoble Alpes, Grenoble, France; b Department of Anesthesiology and Critical Care, Grenoble Alpes University Hospital, F-38000 Grenoble, France; c Univ. Grenoble Alpes, CNRS, UMR 5525, VetAgro Sup, Grenoble INP, TIMC, 38000 Grenoble, France

**Keywords:** Cardiac arrest, Mountain rescue, Chest compressions, Hoisting, Basic life support

## Abstract

**Background:**

Out-of-hospital cardiac arrest (OHCA) in mountainous environments presents substantial logistical challenges, particularly in maintaining high-quality chest compressions during helicopter evacuations. Prolonged interruptions, especially during hoisting, may critically impact neurological outcomes. This study aimed to assess the feasibility and effectiveness of a mechanical chest compression (MCC) device compared to manual compressions during a simulated helicopter hoisting scenario.

**Methods:**

This was a prospective, crossover simulation study involving complete mountain rescue teams. Each team completed two scenarios: one using an MCC device (LUCAS-3®) and one using manual chest compressions. Hoisting was performed at two heights (15 m and 30 m). The primary outcome was chest compression fraction (CCF). Secondary outcomes included, compression depth and rate, and overall safety of the procedure. Results are reported as mean ± standard deviation.

**Results:**

CCF was significantly higher in the MCC group compared to the manual group (96.6 % ± 0.3 vs. 73.9 % ± 6.6; *p* = 0.03). Compression rate was more consistently maintained within recommended ranges (103.0 ± 1.4 cpm vs 136.5 ± 8.7 cpm; *p* = 0.03 ). The guidelines-recommended range for chest compressions was significantly higher with the MCC device (89.5 % ± 9.6 vs 7.5 % ± 6.3; *p* = 0.03). No adverse safety events were observed.

**Conclusion:**

In a simulated mountain rescue setting, the use of a mechanical chest compression device during helicopter hoisting appears feasible, safe and seems to improve chest compression fraction and the rate of guideline-compliant chest compressions.

## Introduction

Out-of-hospital cardiac arrest (OHCA) poses substantial public health challenges, with an annual incidence estimated at 20–170 cases per 100,000 inhabitants^1^ depending on the region. Despite advances in emergency care, survival rates remain low, typically under 8 %.^1^ In mountainous fields, additional challenges such as limited accessibility, extreme environmental conditions, and the requirement for rapid and efficient intervention further complicate emergency response.^2^, ^3^

Mountain sports, widely practiced worldwide, are associated with an increased risk of OHCA, particularly among individuals over the age of 34, who have a 4.3 increased relative risk compared to the general population.^4^ Environmental and physiological factors, such as altitude, hypoxia, and intense physical exertion may contribute to this elevated risk.^5^, ^6^ The presence of bystanders, efficient emergency response systems, and the increasing use of helicopter evacuations are known to improve patient outcomes in these conditions.^7^, ^8^

Mechanical chest compression (MCC) devices are currently recommended for medical cardiac arrest requiring prolonged resuscitation during transportation.^9^ In mountain rescue settings, these recommendations also apply, with interest in cases involving hypothermia or avalanche burial.^10^ However, one of the major challenges in mountain rescue is maintaining continuous chest compressions. Manual compressions are frequently interrupted during helicopter transport, particularly during hoisting, leading to critical periods of hands-off time inducing significant no-flow periods during cardiopulmonary resuscitation (CPR). MCC devices could offer the opportunity to maintain high-quality CPR in extreme conditions.^5^, ^8^, ^11^, ^12^ The feasibility and the benefits of this intervention have been demonstrated even at high altitudes.^5^, ^6^, ^7^, ^8^ In addition, to providing consistent chest compressions, MCC facilitates patient handling, enhances transport safety, and frees up a rescuer for other essential tasks.^13^ One study has shown that using the MCC device during helicopter transport improves the quality of chest compressions and reduces no-flow time, though it may delay the first defibrillation.^14^

To date, while several studies have demonstrated the benefits of MCC in challenging environments^6^, ^7^, ^8^ and in helicopters,^14^ no study has specifically evaluated its use during the hoisting phase of helicopter rescue.

The objective of this study was to assess the feasibility and effectiveness of using an MCC device during the hoisting phase of helicopter rescue evacuation in a simulated mountain rescue, compared to manual chest compressions.

## Methods

### Study design

This prospective crossover simulation study compared mechanical chest compressions (interventional group) with manual chest compressions (control group) during a simulated mountain rescue involving helicopter hoisting. The study was conducted under controlled conditions at the Civil Security base at Versoud Aerodrome (Isère, France), and evaluated the quality of chest compressions both on the ground and during hoisting. It was designed as a pilot, proof-of-concept study, primarily aimed at assessing feasibility and safety before further evaluation in real rescue conditions. Randomization was performed before each experiment by the investigators using a block-of-two draw to assign manual or mechanical chest compression, following the 2025 CONSORT guidelines.^15^

### Primary and secondary outcomes

The primary outcome was the chest compression fraction (CCF), defined as the cumulative duration of effective chest compressions divided by the total duration of cardiopulmonary resuscitation (CPR). CCF was compared between the two groups, both on the ground and during the hoisting phase.

Secondary outcomes included average compression depth (millimeters, [mm]) and average compression rate (compressions per minute, [cpm]). The percentage of compressions performed within the recommended frequency range was also analyzed. In addition, compression performance, expressed as “performed work,” was calculated by multiplying the number of chest compressions by their average depth and dividing by the total duration of the recording.^5^ Feasibility and safety of installing the MCC device into the rescue stretcher were evaluated through identification of key steps and technical difficulties encountered. Operational safety during hoisting was assessed by recording any adverse events, such as uncontrolled rotation, stretcher instability, equipment loss, or other unplanned incidents during the procedure.

### Preparation prior to experiments

**Fig. 1:**
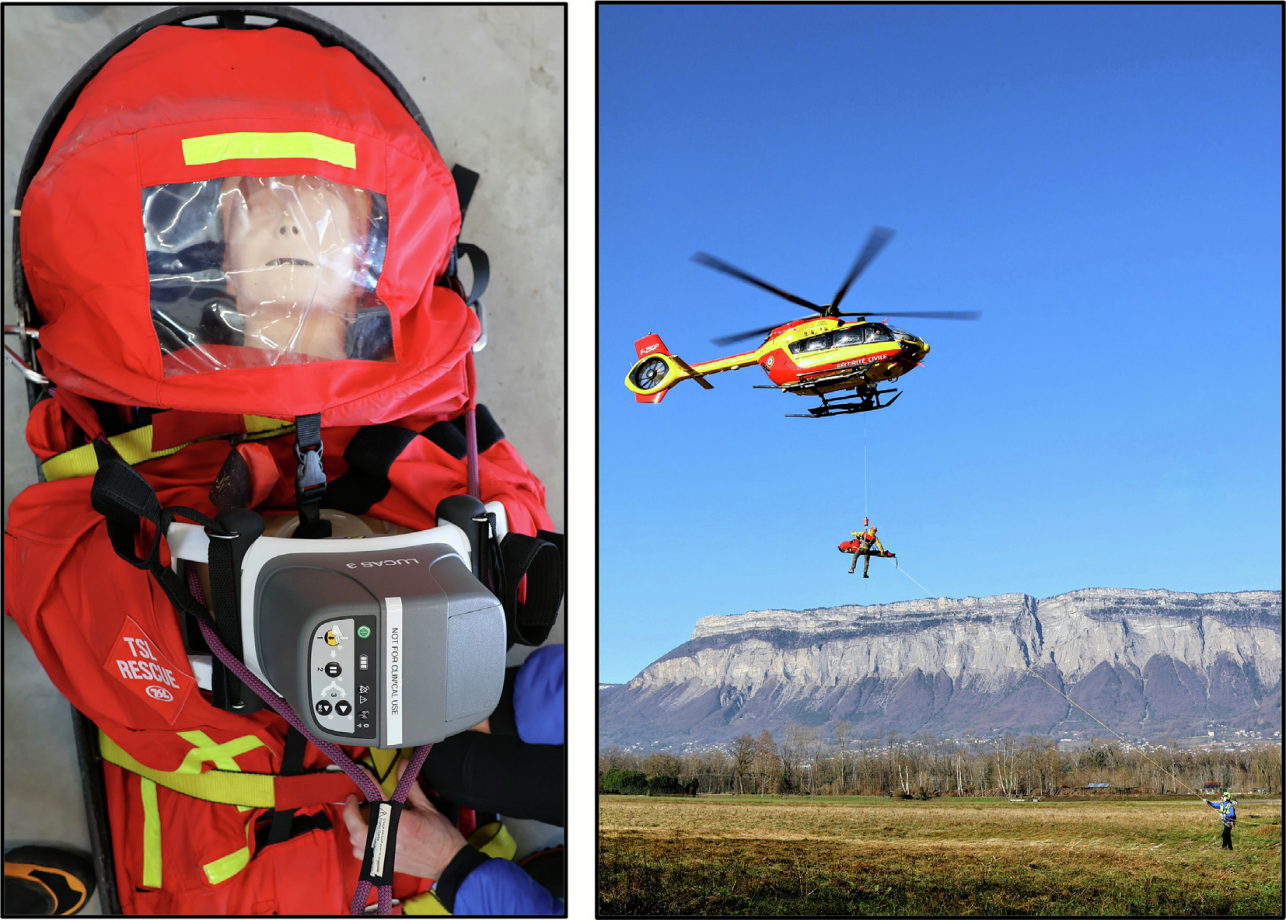
**Equipment and procedural setup used during the simulation: application of the mechanical chest compression device on the manikin in the stretcher (left) and helicopter hoisting phase using anti-rotation rope (right)**.

Prior to data collection, a detailed operational protocol was developed in collaboration with emergency physicians, mountain rescue professionals, and helicopter crew members to standardize procedures and ensure reproducibility. A Laerdal® Resusci Anne QCPR (Norway) was used for this study. Due to the rigidity of the manikin’s arms, they were removed to enable proper installation with the MCC on the hoisting stretcher. The manikin weight (11 kg without arms) was augmented with an anatomically distributed load on the stretcher to approximate a total body weight of 60 kg: 10 kg at the head, 10 kg at the thorax, and 30 kg at the lower limbs (Fig. 1).

### Experimental procedure

Simulations were conducted using full French mountain rescue teams, composed by two specialized rescuers, a helicopter pilot and a flight assistant-hoist operator, with an emergency physician from Grenoble University Hospital. A bystander assisted in placing the manikin into the hoisting stretcher. Each team completed two consecutive scenarios in a crossover design: one using MCC (LUCAS‑3®, Stryker, USA), and one using manual chest compressions (MAN).

Each simulation followed a standardized sequence: initiation of chest compressions on the ground, preparation and loading of the manikin into the stretcher, hoisting (with an anti-rotation line to prevent rotation during hoisting), and the transfer into the helicopter (Fig. 1). In the MCC group, the device was placed before loading and remained active during the entire hoisting process. In the manual group, compressions were stopped during hoisting and resumed once the patient was inside the helicopter. This experiment focused solely on chest compressions and did not include defibrillation, drug administration, or airway management.

In the MCC and MAN group, data collection ended 30 s after helicopter entry. Each simulation lasted approximately 10 min with continuous data recording.

Hoisting was performed at two representative heights (15 and 30 m), with equal allocation between groups. Helicopter and hoisting conditions were standardized within sessions to ensure comparability.

All equipment (manikin, stretcher, MCC device, hoisting system) remained consistent across simulations. Technical steps such as strapping, positioning, and deployment of the anti-rotation rope were strictly standardized to ensure safety and reproducibility. The physician always accompanied the stretcher during hoisting.

### Data collection

Chest compression data were recorded using an acquisition and control system connected with the manikin (SimPad®, Laerdal, Norway). Parameters recorded included compression rate (cpm), depth (mm), percentage of compressions within target range. Data were collected automatically via the SimPad® and manually verified by a clinical research assistant.

### Statistical analysis

Only quantitative variables were analyzed, reported as mean ± standard deviation. To compare the two groups, data were analyzed as non-parametric using a Mann-Whitney *U* test. The same statistical approach was used for secondary outcomes. A subgroup analysis compared outcomes between the two hoisting heights following the same statistical analysis procedure was performed. No correction for multiple comparisons was applied. As this was a feasibility study, no prior sample size calculation was performed. Due to logistical constraints, eight simulations were planned and conducted (four per group).

## Results

A total of eight simulation trials were completed across two days (December 16, 2024 and June 10, 2025). There was no significant difference in mean scenario duration between the control group (manual chest compressions, MAN) and the intervention group (mechanical chest compressions, MCC) (9.4 ± 3.4 min vs. 9.0 ± 0.9 min; *p* = 0.9).

### Primary outcome

**Fig. 2:**
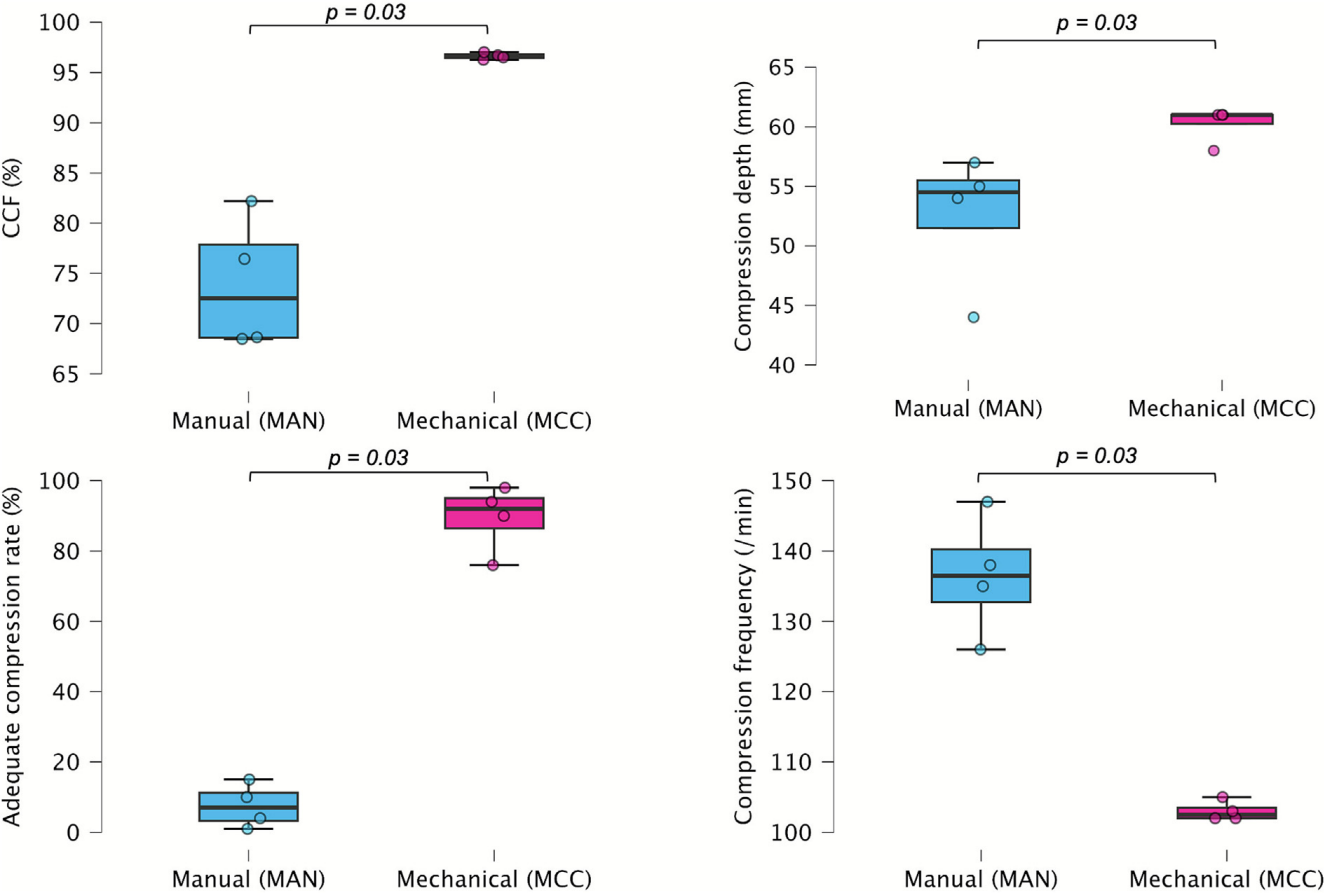
**Box plot of key chest compression quality according to compression type (manual vs. mechanical). CCF: Chest Compression Fraction**.

Chest compression fraction (CCF) was significantly higher with a mean difference of 24.1 % (95 % CI 14.1; 28.5) in the MCC group compared to the MAN group (96.6 ± 0.3 % vs 73.9 ± 6.6 %; *p* = 0.03) (Fig. 2).

### Secondary outcomes

The compression rate was more consistently controlled in the MCC group than in the MAN group (103.0 ± 1.4 cpm vs 136.5 ± 8.7 cpm; *p* = 0.03). Accordingly, the percentage of compressions performed within the guidelines-recommended range (100–120 cpm) was significantly higher with the MCC device compared to the MAN group (89.5 % ± 9.6 vs 7.5 % ± 6.3; *p* = 0.03). The depth of compressions was also greater in the MCC group, with a mean of 60.3 ± 1.5 mm versus 52.5 ± 5.8 mm in the MAN group (*p* = 0.03). There was no statistically significant difference in performed work between groups (7082.3 ± 797.5 in MAN vs 6120.6 ± 149.8 in MCC; *p* = 0.2). All the outcomes are reported in Table 1 and Fig. 2.

**Table 1:** Comparison of chest compression quality parameters between manual (MAN) and mechanical chest compression (MCC). *All statistical analysis were performed using a Mann-Whitney U test.*

**Settings**	**MAN** **(Mean ± SD)**	**MCC** **(Mean ± SD)**	**Mean difference** **(95 % CI)**	***p***
Test duration (min)	9.4 ± 3.4	9.0 ± 0.9	−0.4 (−4.8; 3.9)	0.9
Chest compression fraction (CCF) (%)	73.9 ± 6.6	96.6 ± 0.3	24.1 (14.1; 28.5)	0.03
Number of compressions	974.0 ± 502.7	896.3 ± 94.6	−77.8 (−704.0; 548.0)	0.5
Average compression depth (mm)	52.5 ± 5.8	60.3 ± 1.5	7.8 (0.4; 15.1)	0.03
Average compression rate (cpm)	136.5 ± 8.7	103.0 ± 1.4	−33.5 (−44.2; −22.8)	0.03
Performed Work (PW)	7082.3 ± 797.5	6120.6 ± 149.8	−961.7 (−1954.6; 31.1)	0.2
Compressions at adequate rate (%)	7.5 ± 6.3	89.5 ± 9.6	82.0 (68.0; 96.0)	0.03

### Subgroup analysis stratified by hoist height (15 m vs. 30 m)

There were no significant differences in CPR quality parameters between hoist heights (Table 2). CCF was 86.1 ± 13.5 % in the 30 m group vs 84.5 ± 14.3 % in the 15 m group (*p* = 0.69). No‑flow time did not increase significantly at 30 m (30 m: 1.5 ± 1.4 min vs. 15 m: 1.1 ± 0.9 min; *p* = 0.69). The only significant difference was a longer hoist duration in the 30 m subgroup (30 m: 1.3 ± 0.3 min vs. 15 m: 0.7 ± 0.2 min; *p* = 0.03).

**Table 2:** Subgroup analysis of key chest compression quality parameters between 15 and 30 m of hoisting. *(MAN: manual chest compression, MCC: mechanical chest compression). All statistical analysis were performed using a Mann-Whitney U test.*

**Settings**	**Hoisting 15 m** **(Mean ± SD)**			**Hoisting 30 m** **(Mean ± SD)**			**Mean difference** **(95 % CI)**	***p***
	**Overall**	**MAN group**	**MCC group**	**Overall**	**MAN group**	**MCC group**		
Test duration (min)	8.1 ± 1.2	7.2 ± 1.0	9.0 ± 0.1	10.3 ± 1.8	11.7 ± 3.7	8.9 ± 1.6	2.2 (−1.5; 6.0)	0.69
Chest compression fraction (CCF) (%)	84.5 ± 14.3	72.5 ± 5.6	96.5 ± 0.3	86.1 ± 13.5	75.4 ± 9.6	96.8 ± 0.4	1.6 (−22.4; 25.7)	0.69
Number of compressions	796.8 ± 144.9	700.5 ± 160.5	893.0 ± 11.3	1073.5 ± 440.1	1247.5 ± 658.3	899.5 ± 163.3	276.8 (−290.2; 843.7)	0.69
Average compression depth (mm)	55.3 ± 8.0	49.5 ± 7.8	61.0 ± 0.0	57.5 ± 2.9	55.5 ± 2.1	59.5 ± 2.1	2.3 (−8.2; 12.7)	1.0
Average compression rate (cpm)	119.5 ± 19.7	136.5 ± 2.1	102.5 ± 0.7	120.0 ± 21.0	136.5 ± 14.9	103.5 ± 2.1	0.5 (−34.6; 35.6)	1.0
Performed work	6417.7 ± 603.9	6639.3 ± 947.5	6196.2 ± 10.5	6785.1 ± 905.8	7525.3 ± 475.0	6044.9 ± 210.6	367.3 (−964.5; 1699.2)	0.87
Hoisting duration (min)	0.6 ± 0.2	0.8 ± 0.1	0.5 ± 0.0	1.3 ± 0.3	1.2 ± 0.2	1.9 ± 0.5	0.2 (0.2; 1.0)	0.03
Duration between start of test to start of hoisting (min)	5.6 ± 1.2	4.9 ± 1.5	6.4 ± 0.4	7.4 ± 2.9	8.5 ± 4.3	6.2 ± 0.7	1.7 (−2.1; 5.5)	0.69

### Operational observations

The average hoisting duration did not differ significantly between the two groups (MCC: 0.9 ± 0.5 min vs. MAN: 1.0 ± 0.3 min; 0.69). Similarly, the delay between the start of the scenario and the initiation of the hoisting was comparable between groups (MCC: 6.3 ± 0.5 min vs. MAN: 6.7 ± 3.4 min; *p* = 0.49). No adverse events affecting operational safety were reported. Only one minor incident occurred in the MAN group, involving the opening of the rescue blanket during hoisting, but with no operational consequences. No uncontrolled rotations were observed in the present study.

## Discussion

This study was the first to evaluate the feasibility and efficacy of mechanical chest compressions (MCC) during helicopter hoisting in simulated cardiac arrest scenario during mountain rescue. Our findings demonstrate that MCC is technically feasible and suggests an improvement of CPR quality metrics compared to manual chest compressions during hoisting.

### Clinical and operational benefits

The most notable finding was the significant increase of chest compression fraction and the reduction of no‑flow time when using MCC. For cardiac arrest in mountain conditions, hoist phases can last several minutes, during which manual chest compressions are not feasible. Except in cases of hypothermic cardiac arrest, where intermittent chest compressions can be performed without worsening outcomes,^16^ no-flow time and CCF remains a key determinant of neurological prognosis. The likelihood of a favorable outcome decreases by approximately 13 % for every additional minute without chest compressions.^17^ By ensuring continuous high‑quality chest compressions during hoisting, MCC provides a major clinical advantage.

This study showed that chest compression fraction was significantly higher with MCC. This improvement reflects preserved compression continuity during critical transport phases. Moreover, compression rate and consistency were significantly better controlled with MCC. Performance consistency across teams supports the reproducibility of MCC in such operational settings.

The technical advantages of MCC during mountain rescue are multiple. Importantly, deploying MCC before the hoisting phase and prior to entering the helicopter is essential, as it is nearly impossible to place the device once in flight. Moreover, the use of MCC frees a rescuer previously dedicated to chest compressions, allowing for better redistribution of human resources. In the event of ROSC, the MCC backboard can remain in place, facilitating rapid redeployment of the device in case of re-arrest. Overall, this reduction in operational workload improves team safety and enables greater focus on patient stabilization and environmental risk management, which is particularly critical in Alpine settings.

### Potential operational risks

Autorotation refers to an uncontrolled spinning of the stretcher and patient beneath the helicopter, caused by aerodynamic forces during hoisting. Although no such events occurred in our study, MCC increases wind drag and may exacerbate oscillations during hoisting. To prevent autorotation, an anti-rotation rope was systematically used in all experiments. Without such stabilization, safety during deployment could be compromised in real-world missions. In addition, space constraints inside helicopters highlight the importance of installing the MCC before hoisting when onboard setup is not feasible.

Surprisingly, a previous work showed that first defibrillation could be delayed when deploying MCC during mountain rescue.^14^ To mitigate this risk, cardiac rhythm analysis should precede MCC initiation. Integrating this step into operational protocols with appropriate training is essential.

### Future developments

Mountain rescue operations could be prolonged compared to urban field, due to transit and on-scene longer time. International studies have reported median times from activation to arrival ranging from 3 to 17 min, on‑scene durations of 10 to 45 min, and overall prehospital times exceeding 60 min in most cases, with 44 % lasting over 90 min.^18^, ^19^, ^20^ These lengthy rescue intervals challenge manual CPR endurance, particularly when considering extracorporeal support target windows like ECMO, set at 90 min in France. MCC offers a sustainable approach to maintain high‑quality resuscitation without exhausting rescue teams. MCC utility is further amplified in cases of profound hypothermia, commonly encountered in Alpine environments, where prolonged CPR may be required until extracorporeal rewarming is achieved.

In a recent retrospective cohort study^3^ conducted in mountainous out-slope environments, the leading cause of cardiac arrest was of cardiac origin (51.1 %), followed by traumatic causes (39.7 %). Hypothermia accounted for only 5 % of cases, and respiratory causes for 1.4 %. While MCC is contraindicated in traumatic situations, most cardiac arrests occurring in mountain settings could represent appropriate indications for MCC when prolonged CPR is required, supporting the findings of the present study.

Additional research in real‑world OHCA cases is needed, along with controlled studies evaluating different stabilization techniques and assessing CPR-related injuries. Moreover, aircraft certification regarding MCC usage in flight conditions also warrants further investigations.

### Limitations

This study has several limitations. Due to technical constraints, the number of simulations was determined a priori without a formal sample size calculation. However, given its design as an experimental pilot study, the findings should be interpreted accordingly. As a simulation study, interventions could not fully reflect the logistical and physiological constraints of real-life rescue operations. Although instrumented, the manikin was not fully compatible with the MCC device: its arms had to be removed for installation, and its body weight was artificially increased to 60 kg through distributed ballast. Furthermore, no ventilation or defibrillation was performed, which could have reduced interruptions of chest compressions and may have artificially improved CPR quality by focusing team efforts solely on chest compressions. Additionally, the feasibility of rhythm analysis prior to MCC deployment was not evaluated in this study, although it is a critical safety concern highlighted in previous research.^14^ Only one model of mechanical chest compression device (LUCAS-3) was tested, excluding comparison with other systems, despite performance differences reported in the literature.^5^ Environmental conditions were also limited: only two winch heights (15 m and 30 m) were tested, which may reduce the generalizability of the results. Similarly, all simulations were conducted at an altitude of 200 m, without assessing the impact of high-altitude hypoxia on compression quality, a factor previously documented to influence CPR quality.^8^ Finally, each simulation lasted approximately 10 min and were focused only on chest compression without defibrillation, drug administration and airway management, which allowed us to isolate and focus on the hoisting phase. While this methodological strength enhances the precision of our findings, it excludes other potential interruptions of chest compressions occurring before or after the hoisting sequence, limiting the global clinical interpretation of the data. More studies will be needed assessing different cardiac arrest scenarios. The performed work metric was found to be poorly discriminant in our study, with an artificially elevated value in the MAN group due to an excessive compression rate. While this metric may be useful in comparing different MCC devices,^5^ it is not appropriate for comparing manual versus mechanical chest compressions.

Although this study focused on mountain rescue, the findings may be applicable to other out-of-hospital contexts where hoisting is required, such as maritime rescue. Future research could explore the feasibility of MCC devices in these settings, which share similar logistical constraints in terms of accessibility, transport time, and safety requirements.

## Conclusion

In a simulated mountain rescue setting, the use of a mechanical chest compression device during helicopter hoisting appears feasible and safe. Compared to manual chest compressions, the use of mechanical chest compressions was associated with a higher chest compression fraction and higher rates of guideline-compliant chest compressions.

## CRediT authorship contribution statement

**Alexandre Carron:** Writing – review & editing, Writing – original draft, Methodology, Investigation, Conceptualization. **Vivien Brenckmann:** Writing – review & editing, Validation, Supervision, Resources, Methodology, Conceptualization. **Alexandre Behouche:** Writing – review & editing. **Pierre Bouzat:** Writing – review & editing. **Lilian Barlet:** Writing – review & editing. **Juliette Meyzenc:** Investigation. **Marc Blancher:** Writing – review & editing. **Katell Berthelot:** Writing – review & editing, Resources. **Guillaume Debaty:** Writing – review & editing, Validation, Supervision, Methodology. **Nicolas Segond:** Writing – review & editing, Writing – original draft, Validation, Supervision, Methodology, Formal analysis, Data curation, Conceptualization.

## Declaration of competing interest

The authors declare the following financial interests/personal relationships which may be considered as potential competing interests: Stryker® provided the authors with a mechanical chest compression device (LUCAS-3®) and a cardiopulmonary resuscitation manikin (Laerdal® Resusci Anne QCPR) for the purposes of this study. Stryker® had no role in the design or conduct of the study; in the collection, management, analysis, or interpretation of the data; or in the preparation, review, or approval of the manuscript, including the decision to submit it for publication. The authors declare no other conflicts of interest.
